# Sleep-Dependent Facilitation of Episodic Memory Details

**DOI:** 10.1371/journal.pone.0027421

**Published:** 2011-11-17

**Authors:** Els van der Helm, Ninad Gujar, Masaki Nishida, Matthew P. Walker

**Affiliations:** 1 Sleep and Neuroimaging Laboratory, Department of Psychology and Helen Wills Neuroscience Institute, University of California, Berkeley, California, United States of America; 2 Section of Psychiatry and Behavioral Science, Tokyo Medical and Dental University, Yushima, Bunkyo, Tokyo, Japan; Harvard Medical School, United States of America

## Abstract

While a role for sleep in declarative memory processing is established, the qualitative nature of this consolidation benefit, and the physiological mechanisms mediating it, remain debated. Here, we investigate the impact of sleep physiology on characteristics of episodic memory using an item- (memory elements) and context- (contextual details associated with those elements) learning paradigm; the latter being especially dependent on the hippocampus. Following back-to-back encoding of two word lists, each associated with a different context, participants were assigned to either a Nap-group, who obtained a 120-min nap, or a No Nap-group. Six hours post-encoding, participants performed a recognition test involving item-memory and context-memory judgments. In contrast to item-memory, which demonstrated no between-group differences, a significant benefit in context-memory developed in the Nap-group, the extent of which correlated both with the amount of stage-2 NREM sleep and frontal fast sleep-spindles. Furthermore, a difference was observed on the basis of word-list order, with the sleep benefit and associated physiological correlations being selective for the second word-list, learned last (most proximal to sleep). These findings suggest that sleep may preferentially benefit contextual (hippocampal-dependent) aspects of memory, supported by sleep-spindle oscillations, and that the temporal order of initial learning differentially determines subsequent offline consolidation.

## Introduction

Substantive evidence now indicates a proactive role for sleep in the consolidation of human declarative memory [1]. However, debate continues regarding the specific sleep stages and brain oscillations supporting these modifications and the qualitative nature of this declarative memory benefit [1], [2], [3], [4], [5], [6]. To date, a collection of studies have highlighted the importance of non-rapid eye movement (NREM) sleep in the consolidation-based stabilization of (non-emotional) episodic memories, maintaining or slowing their trajectory of forgetting over time, relative to equivalent time periods spent awake [7].

Beyond sleep stages, associated NREM oscillations, including slow waves and sleep-spindle oscillations, continue to be implicated in the processing of declarative memories [8], [9], [10], [11], [12]. Sleep-spindles, measured with surface electroencephalography (EEG), represent phasic oscillations commonly between 10–16 Hz, persisting for 1–3 s [7], [13]. Consistent with their proposed role in declarative memory, spindles are temporally linked, subcortically, with hippocampal sharp-wave ripple oscillations [1], [14], [15], [16], [17], which may play a causal role in consolidation and the transition from hippocampal to more neocortical memory dependence [18], [19], [20]. Sleep-spindles have further been separated into fast frequency (∼13–15 Hz) and slow frequency (∼11–13 Hz) subtypes [21], [22], associated with unique functional anatomies, with faster sleep spindles being selectively associated with greater activity in, amongst other regions, the hippocampus [23].

While studies to date have productively demonstrated sleep-dependent consolidation of human memory, the qualitative nature and specific components of episodic memories that are modulated by sleep remain largely uncharacterized [24], as does the relationship between sleep-dependent consolidation and the temporal order in which prior information was encoded. Independent of sleep, one paradigm that has helped gain an increasingly nuanced understanding of episodic memory characteristics is the distinction between item memory and context memory [25], [26], [27]. Item memory is suggested to reflect a remembrance of elements of an episodic experience, such as *what* events happened, whereas context memory is considered to involve more relational remembering of the contextual features in which those elements occurred, such as *when* (temporal-order), *where* (spatial) or *how* (source) these events happened [28].

Considerable evidence suggests that these two aspects of episodic memory may rely on related, but distinct, anatomical aspects of the medial temporal lobe. Specifically, neuroimaging as well as lesion studies indicate that item memory can be supported by extra-hippocampal structures, notably the perirhinal and parahippocampal cortices [29], [30], [31], [32], [33], [34], [35]. In contrast, contextual features of episodic memory appear to critically depend on the hippocampus and its integrity, the function of which may be to bind related contextual elements into a contiguous representation [31], [33], [34], [36], [37]. Therefore, the use of a paradigm examining both item and context memory performance allows for both a characterization of qualitative components of declarative memory that are modulated by sleep-dependent processes, and tentative inferences about the potential corresponding neuroanatomy associated with these offline changes. Additionally, such paradigms also offer the ability to examine what, if any, influence the temporal order in which encoding of such information occurs has on the latent offline consolidation [38], [39], [40], [41].

Motivated by this memory framework, and the emerging role of sleep-spindles in hippocampal-dependent information processing, here we test the related hypotheses that (i) sleep (a nap) preferentially benefits hippocampal-dependent aspects of memory representations, facilitating the offline retention of contextual characteristics of episodic experiences, relative to basic item-memory properties, (ii) that such memory benefits correlate not only with NREM sleep amounts, but specifically fast sleep spindle oscillations previously associated with selective hippocampal activity, and (iii) that these memory retention benefits are dependent on the temporal order of initial memory encoding, prior to sleep.

## Methods

### Participants

Twenty-seven healthy adults aged 18–23 years old (mean 20.6 [s.d. ±1.5]; 12 females) participated in the study, keeping a regular sleep schedule for three days prior to the study (7–9 hr sleep, morning rise time between 6:30–8:30am), abstaining from caffeine and alcohol 72 hr prior to, as well as during the study. Exclusion criteria were a history of neurologic, psychiatric or sleep disorders, past history of drug abuse, and current use of anti-depressants or hypnotic medication. The study was approved by the Beth Israel Deaconess Medical Center Institutional Review Board and conducted according to the principles expressed in the Declaration of Helsinki, with all subjects providing written informed consent.

### Experimental design

**Figure 1:**
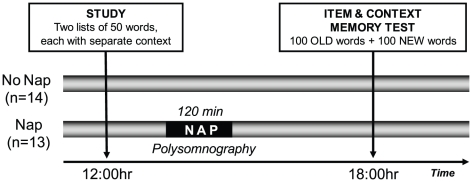
Experimental design. Participants studied two lists of 50 words, one after the other. The two lists were each associated with a different set of contextual cues, making each list distinct. Subjects were then randomly assigned to the Nap- or No Nap-group, with participants in the Nap-group obtaining a 120 min sleep opportunity, while the No Nap-group performed standard daily activities. After 6 hours of offline consolidation time, both groups returned for a memory recognition test, where the 100 old items were presented together with a 100 intermixed foils (new items).

The experimental protocol (**Figure 1**), involved an initial encoding session at 12:00 hr, followed 6 hr later by a delayed recognition memory test session at 18:00 hr. Following encoding (detailed below), participants were randomly assigned to either a Nap-group (*n* = 13, 6 females, mean age 20.5 [s.d. ±1.5]), or a No Nap-group (*n* = 14, 6 females, mean age 20.5 [s.d. ±1.5]). Those in the No Nap-group remained awake across the 6 hr delay, performing their usual daily activities, while those in the Nap condition were allowed a 120 min nap opportunity monitored with polysomnography (PSG) in the sleep laboratory which commenced approximately 45 min after the end of the memory test. After this sleep period, participants in the Nap-group similarly resumed standard daily activities before returning for the recognition memory test at 18:00 hr. Prior to the encoding and recognition tests, all participants completed the Stanford Sleepiness Scale; a standard measure of subjective alertness ranging across a 7-point scale (1 being most alert [42]. One participant in the Nap-group did not fill out Stanford Sleepiness Scales, resulting in *n* = 12 for the Nap group and *n* = 14 for the No Nap-group for the Sleepiness measure.

### Task

#### Encoding

The intentional encoding session was administered in an experimental room on a 14.1″ laptop computer screen at a standard distance from the participant. During encoding, participants studied two separate lists of single nouns (List1 and List2, **Table S1**), with each list associated with a unique contextual cue (a poster), described below. A total of 100 words were selected from the Toronto Noun pool, matched for word frequency, concreteness and word length. The words were then separated equally into two sets (A and B), and used in a counterbalanced order across participants assigned as either List1 or List2. As in previous studies [30], [31], [43], [44], [45], [46], [47], context was enforced using a strong visual cue, with the two separate word lists each being associated with a different detailed visual poster in front of participants, with the instruction for participants to associate each word on the screen with the poster in front of them. Specifically, participants faced one wall of the experimental room during encoding of the first list, with the unique detailed poster placed at eye height. Participants were then turned 180 degrees and sat on the opposite side of the room and encoding the second list facing a different unique poster placed at the same eye height). One poster depicted the cartoon characters of “the Simpsons” and one poster depicted a scene with two children and a surreal background (specific poster detail provided in **Figure S1A** and **S1B**). To further confer unique context to learning of each of the two lists, additional supplemental contextual cues were enforced: *screen location* (either upper left quadrant presentation or upper right for each list) and *font color* (either blue or red, both on a white background for each list). The two lists were counterbalanced across participants in terms of assignment to these contextual manipulations.

The encoding session started with an example trial to instruct participants on how to form an association between the poster and the item. The example trial presented the word item ‘*book*’ and participants were given the example association of “*The Simpsons never read books*”. Following the example trial, the encoding session began with List1. For each list, each trial started with a fixation cross for 50 ms in the upper left or upper right quadrant of the screen corresponding to the target presentation location of the ensuing word, after which a single word was presented at that location in lowercase Courier New font size 25 for 4000 ms. Next an instruction screen appeared, informing the participant to press the space bar after they had successfully associated the word with the poster in front of them (with response times logged), followed by a blank screen that appeared for 2000 ms, after which the next trial would begin. It should be noted that no differences in the average time participants spent on associating the word on the screen with the contextual cues were present between the No Nap-group ([mean ± s.e.m.] 3961±651 msec) and the Nap-group (4930±866 msec), unpaired *t*-test *t* = 0.90, *p* = 0.38). Each of the two lists consisted of 50 words (50 trials), and each word was presented only once. Learning of the two lists was separated by a 3 min break.

#### Recognition Test

As with encoding, the recognition test session was similarly administered on a 14.1″ laptop computer screen at the same a standard distance from the participant as encoding, and in the same room. However, for the recognition test, participants were oriented 90 degrees to either of the encoding directions in the room, with the original wall posters previously informing context during encoding removed. During recognition testing, all 100 previously studied words were presented in random order, together with 100 randomly intermixed new words (foils), also selected from the Toronto Noun pool, similarly matched for word frequency, concreteness and word length to the studied word set.

Each recognition trial began with a word appearing in the middle of the screen in black lowercase Courier New font size 25 on a white background with the two response options listed below the word. During this self-paced screen participants first indicated whether they believed the stimuli to be ‘old’ (from either of the two study lists) or ‘new’ (not seen before) using keyboard responses, providing a measure of item-memory. After the response the word was cleared from the screen. Second, if participants responded ‘new’, the next recognition trial began, yet if participants responded ‘old’, a subsequent contextual memory decision was made by participants, indicating which of the two lists and associated contexts the word came from. For this context decision, digital images of the wall-posters were presented in the middle of the screen one at a time for 1250 ms each to designate the two context/list choice options, after which participants indicated which list the remembered the word came from; the first list studied (List1), or the second list studied (List2), providing the measure of context-memory. Both the item- and context-memory choice-response phases were self-paced. During the contextual memory component of the trial the word itself was not on the screen.

In addition to the memory test at 18:00, participants additionally performed a two-alternative forced choice reaction time task, which served as an alertness response measure following either the Nap or No Nap experimental manipulations. This commonly used reaction time metric offered an objective alertness measure that complimented the subjective Stanford Sleepiness Scale [48], [49], [50], [51], [52]. The task consisted of 18 trials. On any one trial, either a “0” or “1” was presented on the screen, with participants required to accurately press the corresponding correct keyboard button as quickly as possible, with the next trial not continuing until participants made a response to the target cue. Equal numbers of each trial type (“0” or “1”) were presented in a randomized order.

### Polysomnographic recording and analyses

Polysomnography (PSG) recording was performed in accordance with standardized techniques (Rechtschaffen and Kales 1968), using digital electroencephalography (EEG), electromyography, and electrooculography signals, acquired with a Grass Colleague system (sampling rate: 256 Hz, high- and low-pass filter 0.3 and 35 Hz, respectively, notch filter 60 Hz). A mastoid referenced PSG electrode montage was utilized, composed of EEG sites F3 and C3 (referenced to A2), and F4 and C4 (referenced to A1). Each sleep epoch was scored in accordance with standard criteria (Rechtschaffen and Kales 1968), blind to participants' behavioral task performance.

### Sleep spindle analysis

Upon removal of waking epochs and movement/muscle artifacts from sleep recordings, sleep spindles analysis focused on NREM epochs, including all electrodes sites, using an automatic algorithm in Matlab (The MathWorks Inc, Natick, MA). Artifacts in the time series were removed by visual rejection, and the raw EEG was first band-pass filtered using a linear finite impulse response (FIR) filter (EEGLAB toolbox [http://www.sccn.ucsd.edu/eeglab/]) into either fast (13–15 Hz) spindle or slow (11–13 Hz) spindle [53], [54] bands, similar to previously reported fast and slow spindle analyses [21], [22]. Specifically, the eegfilt function in EEGlab was used, creating FIR filters corresponding to the low and high pass frequency characteristics desired and the sampling rate of the data (256 Hz). For the current study this yielded high pass filters at 11 and 13 Hz (with orders of 69 and 57 respectively) as well as low pass filters at 13 and 15 Hz (with orders 57 and 51 respectively). These low and high pass filters were used in conjunction to band pass filter the data into slow and fast spindle ranges, respectively. Spindle density was evaluated using a validated automated EEG spindle detection algorithm, developed by Tononi and colleagues [53], [55]. In short (but for details see [53]), the amplitude of the rectified signal was used as a unique time series, identifying amplitude fluctuations exceeding threshold values, with the lower and upper values set at two and eight times the average amplitude. The algorithm-determined spindles were restricted only to those events falling within the specific frequency range. Two participants had excessive channel artifact precluding reliable spindle estimation, resulting in *n* = 11 for spindle analyses.

### Analysis of memory performance

For the item-memory measure, the old/new choice resulted in 4 possible response classifications: correct old judgments (‘hits’), incorrect old judgments (‘misses’), correct new judgments (‘correct rejections’), and incorrect new judgments (‘false alarms’). Item recognition memory accuracy (d′) was calculated according to signal detection theory i.e. the difference between the z-transformed (normalized) probabilities of hit and false alarm rates: d′ = z(hit rate)−z(false alarm rate) [56]. For the context-memory measure, the contextual judgment (first list or second list) yielded either a correct or incorrect outcome, indexed as the proportion correct, calculated by dividing the correctly answered items by the total number of context-memory items, resulting in a score between 0 and 100. Planned comparisons were performed for item- and context-memory between the two groups, as well as for List1 and List2 separately, to investigate the influence on temporal order of encoding.

## Results

### Memory performance and sleep stage associations

**Figure 2:**
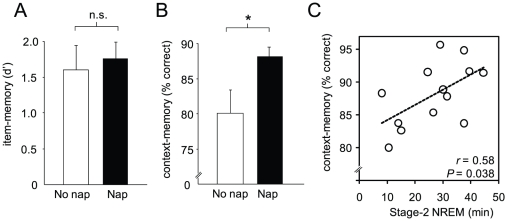
Memory performance and sleep association. A) Item-memory performance and B) context-memory performance for the No Nap- (clear bar) and Nap-group (filled bar). C) Within the Nap-group (filled bar in 2B) the extent of context-memory retention significantly correlated with total time spent in stage-2 sleep. Error bar represents s.e.m. * *P*<0.05 n.s. = non-significant.

At recognition testing, for the measure of item-memory, no difference in offline retention was observed between the No Nap-group ([mean ± s.e.m.]: 1.85±0.28) and Nap-group (1.95±0.20, unpaired *t*-test *T* = 0.30, *P* = 0.77; **Figure 2A**), yet in contrast, a significant between-group difference was observed for context-memory (**Figure 2B**). Specifically, a selective retention benefit in offline context-memory was observed following sleep in the Nap-group (0.88±0.01) compared to the No Nap-group (0.80±0.03, unpaired *t*-test: *T* = 2.14, *P* = 0.04; **Figure 2B**). Therefore, while a memory retention advantage was conferred by sleep (albeit a nap) relative to wake, this advantage was selective for the contextual qualitative features of the prior encoding episodic representations, rather than the basic item memory retention.

Next, to examine the relationship between context-memory performance following sleep and sleep stages in the Nap-group, sleep-stage values were correlated with memory performance (values represented in **Figure 2B**, filled bar). The sleep-stage values of the nap are summarized in **Table 1**. Within the Nap-group, the extent of context-memory retention was positively correlated with the amount of stage-2 NREM sleep obtained (*r* = 0.58, *P* = 0.038, **Figure 2C**). No other stage of sleep (stage-1 NREM, SWS or REM), nor total sleep time, correlated with context-memory performance (**Table 1**). Complementing the lack of between-group difference in item-memory performance, and further indicative of specificity, no significant sleep-stage correlations with item-memory were observed (all *P*>0.48, **Table 1**). Therefore, the memory characteristic demonstrating superior performance in the Nap-group relative to the No Nap-group – context-memory – was additionally and selectively predicted by the amount of intervening Stage-2 NREM obtained in the Nap-group.

**Table 1:** Polysomnography sleep-stage values for the Nap-group (mean ± SEM).

	Sleep Time (min)	% Sleep Time	Obtained by % of Participants	Item memory	Context memory
Total nap time	97.9±5.4			0.13	0.26
Stage 1	23.4±4.4	26.1±5.4	100%	−0.06	−0.18
Stage 2	26.8±3.3	27.1±3.0	100%	0.21	0.57*
SWS	37.3±6.3	36.7±5.4	92%	0.14	0.04
REM	10.4±2.3	10.1±2.2	85%	−0.26	0.03

Mean duration (in minutes), percent (%) and standard error (SEM) of total nap time, NREM sleep stages 1 and 2, slow-wave sleep (SWS; NREM stage 3 & stage 4) and rapid eye movement sleep (REM) along with Pearson correlation values between minutes spent in each stage and item- and context-memory performance.

*P<0.05.

### Memory performance and sleep-spindle associations

**Figure 3:**
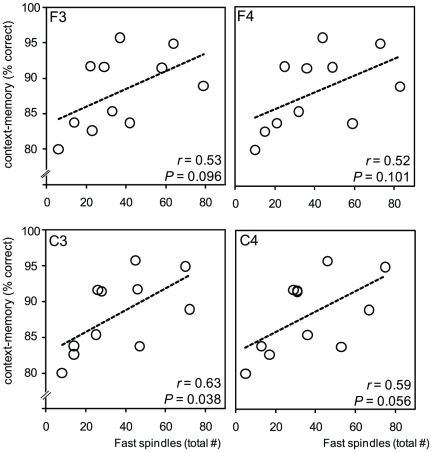
The association in the Nap-group between context-memory retention and fast sleep spindles across the four electrode derivations (left top corner box label), with corresponding *r*- and *P*-values provided.

Building on this selective stage-2 sleep correlation, and our *a priori* predictions, we next tested the hypothesis that the extent of context-memory retention in the Nap-group would be proportional to the number of sleep spindles – the hallmark oscillation of stage-2 NREM – and specifically the number of fast spindles, due to their association with hippocampal activity [1], [19], [23]. Supporting this prediction, the degree of context-memory retention following sleep positively correlated with stage-2 NREM fast spindles across all electrode sites, being most significant over the left central derivation (C3: *r* = 0.63, *P* = 0.038; **Figure 3**). This effect was not present for slow spindles (all *r*<0.40, all *P*-values>0.23, **Table S2**). Therefore, beyond the predictive relationship with a specific sleep-stage (stage-2 NREM), the number of fast sleep spindles during this brain state additionally correlated with the extent of context-memory retention following sleep.

### Selective order effects in context-memory retention and sleep associations

Finally, and based on emerging evidence for differential consolidation based on the temporal order of learned material during initial encoding [38], [39], [40], [41], we sought to determine whether the offline retention of context-memory was related to the order in which the initial lists were learned: first (List1) or second (List2). Therefore, we analyzed performance and sleep associations for each list separately.

**Figure 4:**
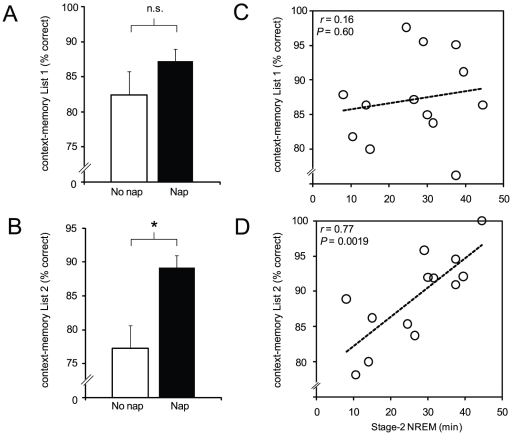
Context memory and sleep association. A) Context-memory performance in the No Nap- (clear bar) and Nap-group (filled bar) for List1, and B) for List2, C) Within the Nap-group, the association between the amount of Stage-2 NREM sleep and the extent of List1 context-memory retention, and similarly D) the corresponding association for List2 retention. Error bar represents s.e.m. *P = 0.028, n.s. = non-significant.

Contrary to the between-group differences described above when performance for both lists were combined, when separated for List1 only (the first list learned), no significant difference in context-memory retention was observed between the Nap- and No Nap-groups (Nap [mean ± s.e.m.]: 0.87±0.02, No Nap: 0.82±0.03, unpaired *t*-test *T* = 1.24, *P* = 0.22, **Figure 4A**). Instead, a significant context-memory retention advantage was only identified for List2; the list encoded second, after List1, and hence most proximal to the sleep (or wake) offline period (Nap [mean ± s.e.m.]: 0.89±0.02, No-Nap: 0.77±0.05, unpaired *t*-test *T* = 2.33, *P* = 0.03, **Figure 4C**). It should be noted that this effect was specific for context-memory considering the two groups were not significantly different in item-memory split per List1 (d′ Nap [mean ± s.e.m.]: 1.92±0.2, No Nap: 1.88±0.3, unpaired *t*-test *T* = 0.11, *P* = 0.92) nor List2 (d′ Nap [mean ± s.e.m.]: 2.05±0.2, No Nap: 1.83±0.3, unpaired *t*-test *T* = 0.65, *P* = 0.52).

**Figure 5:**
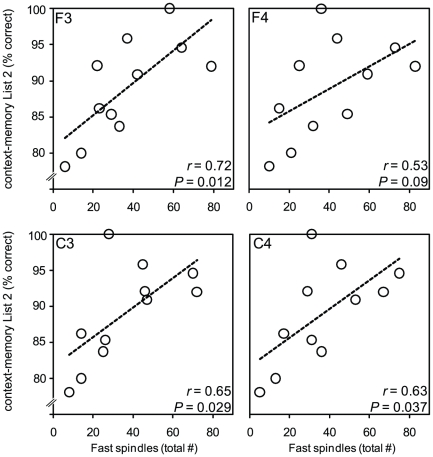
The association in the Nap-group between context-memory retention for List2 and fast sleep spindles across the four electrode derivations (left top corner box label), with corresponding *r*- and *P*-values provided.

Finally, consistent with this selective context-memory order-effect, a highly significant correlation was identified between context-memory retention for List2 information in the Nap-group and prior stage-2 NREM sleep (*r* = 0.77, *P* = 0.002, **Figure 4D**), yet no such association between context-memory for List1 information and stage-2 NREM was observed (*r* = 0.16, *P* = 0.60, **Figure 4B**). Similarly, strong and significant correlations between fast sleep spindles and context-memory for List2 were observed within the Nap-group, particularly over the F3 electrode site, proximal to the left prefrontal cortex (**Figure 5**). No significant associations between fast sleep spindles and context-memory retention of List1 were evident (**Figure S2**). Furthermore, no significant correlations between slow sleep spindles and context-memory for List1 were identified (all *r* values<0.16, all *P*-values>0.65; **Table S2**). Several, albeit weaker correlations were evident between slow sleep spindles and List2 context-memory (**Table S2**). Other fast sleep spindle measures such as density, frequency, duration and sigma power were also examined. None correlated significantly with contextual memory performance for either of the two lists (all *r*<0.37; *P*>0.27) or List2 (*r*<0.44; *P*>0.18).

Taken together, these data indicate that the overall advantage for context-memory in the Nap-group relative to No Nap-group appeared to be driven principally by an advantage conferred to information from List2 (encoded second), and not List1 (encoded first), with preferential associations with stage-2 NREM sleep and fast sleep spindles also seen for List2. Therefore, the benefit of sleep on context-memory was sensitive to the temporal order in which the information was learned, such that only information learned last, prior to sleep, demonstrating a significant retention advantage between the two groups following sleep.

It is important to note that fast spindle count correlated with stage-2 sleep time (average of all electrodes: *r* = 0.66), making it challenging to determine whether fast spindles or the stage of sleep from which they emerge are principally influencing memory performance. Fast sleep spindle density—a measure that normalizes sleep time—also positively correlated with List2 context-memory performance, albeit not significantly (*r* = 0.37, *P* = 0.26). However, that the relationship between fast spindles and context-memory varies across the different topographical locations, and is not simply dependent on the total count of fast spindles at any electrode site, offers support for a role of spindles in regional memory processing. Specifically, while the F3 channel expressed fewer fast spindles than the F4 channel (F3 mean: 37; F4 mean: 41), the F3 site demonstrated a stronger correlation with memory performance that the F4 site (F3: *r* = 0.72; F4: *r* = 0.53), despite spindle counts for each channel being derived from the same total stage-2 time. These data suggest that while the two measures are co-linear, fast spindles contribute additional explanatory information, suggesting the location of the fast spindles (beyond simply total number per se) predicts context-memory retention.

### Response bias

The test of temporal memory used in the current study has the potential limitation of being influenced by bias (response tendency) for one of the two lists. Therefore, the possibility of differences in response bias (List1/List2) between the two groups was examined by assessing the response rates of both lists to New items, and specifically New items that were incorrectly labeled as ‘Old’. In these instances, participants still had to make a decision as to which list (List1/List2) the item came from (the context-memory judgment). As such, these specific items offer an opportunity to examine the presence of any outright bias in contextual responses to novel items to which participants were not previously exposed, and that have not passed through the brain state of wake or sleep. Contrary to a bias hypothesis between the two groups, no significant difference was observed in the rate of List1 and List2 responses to these New items (proportion of List 1 responses relative to List 2: mean [s.e.m.] Nap-group: 0.62 [0.06] and No Nap-group: 0.61 [0.03], *P* = 0.86). These analyses suggest that the context-memory differences reported between the two groups are not parsimoniously accounted for on the basis of bias.

### Sleepiness Scales, Sleep Logs and Alertness

On the night prior to the experimental day, sleep logs demonstrated both groups obtained a similar amount of sleep the night prior to learning: No Nap-group 8.1 hr (s.d. ±1.1), Nap-group obtained a mean of 7.5 hr (s.d. ±1.0; unpaired *t*-test *P* = 0.15). The average scores on the Stanford Sleepiness Scale (**Table 2**) also did not differ between the No Nap-group and Nap-group at the initial encoding session, (mean 2.1 s.d. ±0.6, mean 2.7 s.d. ±1.3, respectively; unpaired *t*-test *P* = 0.17). A non-significant trend in this measure was observed between the Nap- and No Nap-group at later recognition test session (mean 1.8 s.d. ±0.7, mean 2.6 s.d. ±1.2, respectively; unpaired *t*-test *P* = 0.06). One concern is that performance differences between the groups at this later recognition test session due, in part, to the state of sleepiness. This possibility did not appear to be parsimonious, however, since neither item- nor context-memory performance correlated with these sleepiness scores in either group (Nap item: *r* = 0.13; Nap context: *r* = −0.12; No Nap item: *r* = 0.28; No Nap context: *r* = 0.31, all *P*>0.28). Data from the objective two-alternative forced choice response-time task also performed at the recognition test session in both groups showed no significant differences in speed of responding between the Nap-group ([mean ± s.e.m.]: 640±25 ms) and No Nap-group (693±52 ms, *T* = 0.90, *P* = 0.37). While this collection of data does not dismiss the impact of alertness on cognitive functions such as memory recollection, it does not appear to support an alertness account for the selective differences in memory retention observed between groups, or within the Nap-group, the sleep-stage and spindle oscillation correlations.

**Table 2:** Sleepiness values in both conditions.

	Encoding	Memory test
No Nap-group	2.1±0.6	2.6±1.2
Nap-group	2.7±1.3	1.8±0.7

Stanford Sleepiness Scale values (mean ± SD) at the time of list encoding (12:00 hr) and the recognition memory test (18:00 hr).

## Discussion

When taken together, our findings demonstrate that (i) a short sleep period (nap) preferentially benefits more hippocampal-dependent aspects of declarative memory representations, promoting superior retention of contextual episodic memory characteristics, relative to basic item-memory properties, (ii) these contextual memory benefits correlate not only with a particular stage of prior NREM sleep (stage-2), but a hallmark electrophysiological EEG oscillation of this sleep stage: fast sleep spindles, and (iii) this contextual memory advantage is dependent on the temporal order in which the initial information memory sets were encoded prior to sleep.

### Qualitative differences in memory retention

An extensive literature now implicates sleep in the consolidation of episodic memory, slowing the decay of forgetting. Such studies have demonstrated that individual episodic item memories and item-item associations are preferentially stabilized during offline periods containing sleep, relative to time awake [1], [57]. While an episodic experience is composed of such individual item details, critical to instantiating an episodic memory, they do not by themselves represent the holistic sum of an episodic experience. An important additional feature sub serving the ensemble that creates an episodic memory is context. The context within which item memory components are created provides an important defining trait of an episodic experience, aiding in the binding or configuration of item elements into a thematic episode [36]. While sleep is known to support the consolidation of individual item memories, its role in modulating these more qualitative features of episodic experiences remains poorly characterized [24]. Our current findings offer insights into the nature of sleep-dependent episodic memory processing, first at the behavioral level, indicating superior offline retention of contextual aspects of episodic memory representations following sleep, at least a nap, relative to basic item-memory. Moreover, such a qualitative dissociation may inform the neuroanatomical mechanisms underlying sleep-dependent memory processing. The hippocampus has consistently been demonstrated to be necessary in the successful binding of contextual components of an experience into episodic memory, while item details of such an experience appear less critically dependent upon hippocampal integrity [28]. This would suggest that aspects of episodic experiences most sensitive to sleep-dependent memory processing may be those that rely most significantly on the hippocampus [58]; a hypothesis further supported by the associations identified with sleep physiology.

### Sleep-stage and sleep spindle association

In addition to a between-group difference in context-memory, within the Nap group, a predictive relationship between this hippocampal-dependent memory measure and the amount of intervening stage-2 NREM, as well as associated fast sleep spindles, was identified. These associations build on a growing collection of reports implicating NREM sleep-spindles in memory processing, describing learning-dependent increases in spindles following initial memory encoding [8], [54], [59], [60], [61], [62], together with predictive spindle correlations (often topographic) and the success of post-sleep memory retention [9], [61], [63], [64], [65], [66], [67], [68].

Mechanistically, the neurophysiology associated with sleep spindles appears especially amenable to the (re)processing of hippocampal-dependent information, such as contextual memory. The expression of surface spindles commonly measured with EEG are temporally linked, subcortically, with sharp-wave ripple events in the hippocampal formation [1], [7], [69], the activity of which is proposed to play a role in hippocampal-neocortical memory interaction [1], [9], [10], [11], [12], [16]. Indeed, sharp-wave ripple events, co-occurring with the cortical expression of sleep spindles, have recently been demonstrated to play a causal role in hippocampal-dependent memory consolidation, critical for promoting the long-term retention of spatial maze learning [19]. Moreover, human neuroimaging reports have established that sleep spindles, and specifically fast-, relative to slow-spindle oscillations, are selectively associated with hippocampal activation [23], [70]. Such hippocampal-related physiological processes would appear to represent one potential explanation underlying the selective fast sleep-spindle relationship we identified with the superior offline retention of contextual memory in the Nap-group. It is important to note that hippocampal activity was not measured in the current study, and confirmation of its direct involvement will require methods that localize functional activity to the hippocampus. Nevertheless, our findings are in line with a selective sleep-dependent effect on hippocampal-dependent memories, predicting increased contextual memory after sleep compared to continued waking experience.

Previous studies investigating sleep and declarative memory have found a similar positive correlation between memory and total spindle count [9], [62], [71], [72], while others instead found spindle density to better predict memory [8], [10], [54]. One concern with total spindle count as opposed to spindle density regards the co-linearity between the total count of fast spindles and the total time spent in stage-2 sleep. However, a process such as memory consolidation is not necessarily expected to correlate with sleep oscillations uniformly across the head. Thus, a measure such as spindle count that can vary across the head provides a better explanatory feature than stage-2 alone. Consistent with this notion, while stage-2 was indeed co-linear with fast spindles in the current study, the relationship between fast spindles and contextual memory varied across different topographical EEG electrode sites, and was not simply dependent on the total count of fast spindles. Nevertheless, the co-linearity of these two measures remains one limitation of the current study. Future work is required that causally manipulates spindle amount while keeping stage-2 time constant in order to confirm a selective role of spindles, beyond the state from which they come.

Although the current study did not identify significant between group differences in item-memory, or within the Nap-group, an association between item-memory (or context) and NREM sleep, this is not to suggest that sleep, or NREM sleep and its oscillations in particular, play no role in episodic memory processing. Slow-wave sleep has consistently been implicated in consolidation of item-memory [11], [73], [74], [75], [76], [77]. At least two factors may explain the lack of an identified slow-wave sleep relationship in the current study. First, the duration of the sleep epoch employed in the current study was not a full night, but rather, a shorter daytime interval. While participants did obtain SWS, the amount (or number of cycles) may be insufficient to produce a robust item-memory effect and hence association. Second is the method of memory test used. A collection of reports describing individual item-memory associations with SWS properties have used tests of free recall (for reviews, see [1]), while the current study utilized a recognition memory paradigm. The different task demands imposed by free recall, relative to recognition, including concepts of accessibility (existing representations that may or may not be accessible) relative to availability (the presence or absence of a representation), may be another feature mediating sleep sensitivity. Studies that examine multiple components of episodic representations within the same experiment, and assessed using different tests of memory, will be required to more clearly dissociate such influences and hypotheses.

### Order effects

When comparing memory performance on the basis of list order – encoded first (List1) or encoded second (List2) – a dissociation was observed. The difference in context memory between groups was principally driven by performance on List2, and not List1 (although it should be noted that the same directional effect was present for both lists). Nevertheless, the detriment in context-memory across wake, relative to the preservation following sleep, was significant only for information learned last (List2), potentially indicative of interference. Inferior memory performance due to proactive interference (PI; as a result of previously having learned similar material) and retroactive interference (RI: as a result of subsequently learned similar material) are long-standing phenomena in theories of forgetting [78], [79], [80]. While both may be contributing to the observed effects in the current study, PI appears to be a more likely candidate, since RI caused by learning of List2 would predict between-group differences in List1 performance, while the opposite was found. A PI account of the current findings instead suggests that subsequent learning of List2 would be compromised in either quantity (e.g. amount of encoding information) or quality (e.g. strength of encoded representations), rendering List2 more vulnerable than List1 to forgetting during the offline delay. The benefit of sleep would, therefore, preferentially preserve List2 information, strengthening these less robust representations; a prediction that is congruent with our current findings. Such a notion would also explain the preferential correlations within the Nap-group between both stage-2 sleep and fast sleep spindles for List2, and not List1. These selective associations are similarly indicative of a role for sleep in the preservation of more vulnerable memory representations, possibly established by PI, and complement evidence that sleep, relative to wake, preferentially enhances more weakly encoded representation over those more strongly encoded [81].

A PI account is not the only explanatory candidate. Rather than an active interference model, a simple proximity theory – contextual information encoded most closely in time to the onset of sleep is preferentially consolidated – could play a role. The timing of the onset of sleep, relative to the completion of encoding, has been demonstrated as important for successful consolidation in some studies [82], [83]. However, this proximity account appears to be a less tenable explanation of our current findings since the specific sleep stage and spindle correlations found within the Nap-group suggest that while there is less interference after List2 than List1 due to the nap, there is an active benefit of sleep that further enhances memory performance for List2. While the current findings alone do not provide a full mechanistic account of this list effect, they do suggest that the temporal order in which contextual information is learned can influence the trajectory of subsequent forgetting across offline wake and sleep states.

In summary, here we show that sleep, relative to a period of daytime wake, selectively strengthens more hippocampal-dependent, contextual aspects of episodic events. Our results further clarify sleep's role in memory processing, demonstrating that sleep modulates contextual memory through specific electrophysiological oscillations consistent with a model of hippocampal-neocortical memory interaction during sleep.

## Supporting Information

Figure S1 **Poster stimuli.** A) One of the two posters participants made associations with during the context-memory encoding task. B) One of the two posters participants made associations with during the context-memory encoding task.
(DOCX)

Figure S2 **The association in the Nap-group between context-memory retention for List1 and fast sleep spindles across the four electrode derivations (top corner box label), with corresponding** ***r*** **- and** ***P*** **-values provided.**
(DOCX)

Table S1 **The two word lists (List1 and List2) consisting of single nouns.**
(DOCX)

Table S2 **Pearson correlation values between contextual memory score for both lists (first row), first and second list and the number of slow spindles recorded at each of the four electrode sites.** *P<0.05, ** *P*<0.03.
(DOCX)
