# Supplementary figures and images for: Sleep-Dependent Facilitation of Episodic Memory Details
Source: PLoS One. 2011 Nov 17;6(11):e27421. doi: 10.1371/journal.pone.0027421 (PMC3219667; doi:10.1371/journal.pone.0027421)

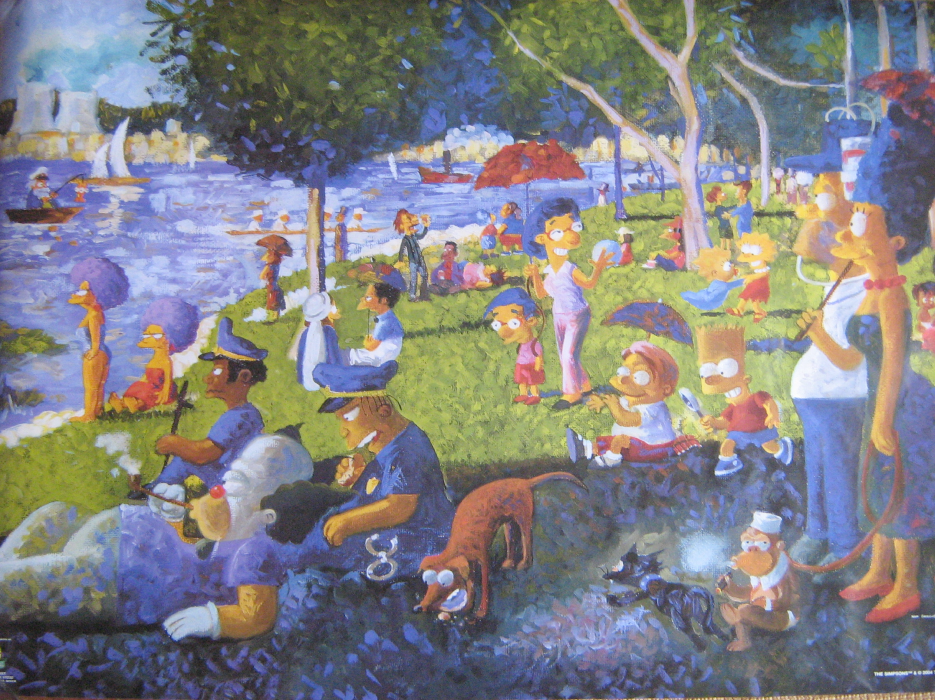


Figure S1A


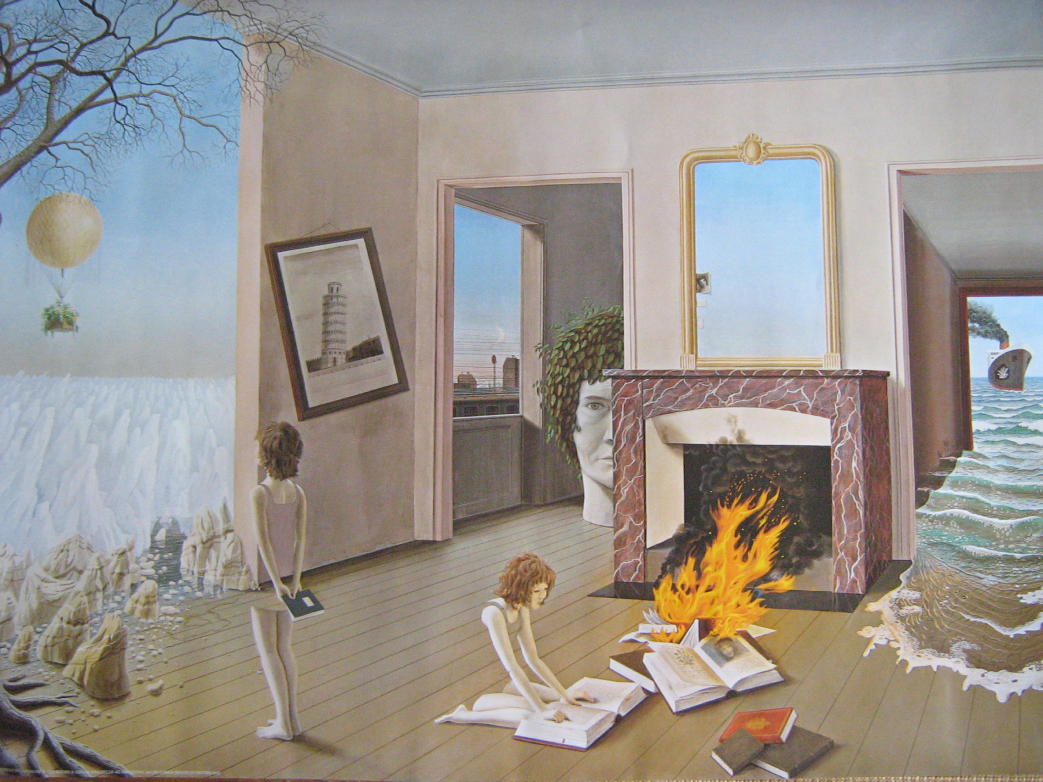


Figure S1B

Supplement: Figure S1 — Poster stimuli. A) One of the two posters participants made associations with during the context-memory encoding task. B) One of the two posters participants made associations with during the context-memory encoding task. (DOCX) [file pone.0027421.s001.docx]

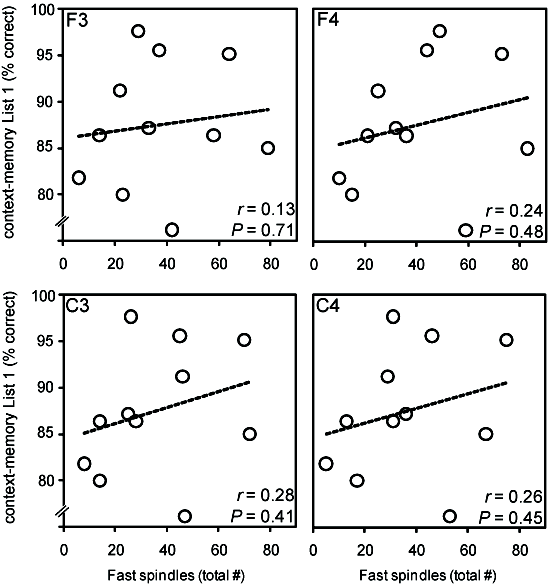


Figure S2

Supplement: Figure S2 — The association in the Nap-group between context-memory retention for List1 and fast sleep spindles across the four electrode derivations (top corner box label), with corresponding r - and P -values provided. (DOCX) [file pone.0027421.s002.docx]
