# Supplementary material for: Sleep-Dependent Facilitation of Episodic Memory Details
Source: PLoS One. 2011 Nov 17;6(11):e27421. doi: 10.1371/journal.pone.0027421 (PMC3219667; doi:10.1371/journal.pone.0027421)
Supplement: Table S1 — The two word lists (List1 and List2) consisting of single nouns. (DOCX) [file pone.0027421.s003.docx]

Table S1: Word lists

| List 1 | List 1 con’t | List 2 | List 2 con’t |
| --- | --- | --- | --- |
| compound  service  future  herald  nation  valley  detail  figure  pupil  merchant  railway  ribbon  clothing  million  image  window  shelter  traffic  pleasure  leather  barrel  river  harvest  bubble  section | jersey  saddle  penny  daylight  parent  reason  training  marriage  success  minute  payment  disease  frontier  carbon  worker  contents  flavor  boundary  tribute  value  account  writing  judgment  learning  contract | carriage  rabbit  body  member  record  master  platform  hatred  mercy  illness  organ  array  congress  story  manner  couple  career  chairman  senate  silence  theater  bedroom  blanket  merit  silver | genius  habit  circuit  vapor  outline  layer  timber  center  corner  justice  circle  garden  painting  fortune  devil  dealer  anchor  scholar  level  total  status  cattle  channel  lecture  vessel |
