# Supplementary material for: Sleep-Dependent Facilitation of Episodic Memory Details
Source: PLoS One. 2011 Nov 17;6(11):e27421. doi: 10.1371/journal.pone.0027421 (PMC3219667; doi:10.1371/journal.pone.0027421)
Supplement: Table S2 — Pearson correlation values between contextual memory score for both lists (first row), first and second list and the number of slow spindles recorded at each of the four electrode sites. *P<0.05, ** P<0.03. (DOCX) [file pone.0027421.s004.docx]

Table S2: Correlations between context-memory score and the number of slow spindles recorded at each of the four electrode sites

|  | **C3** | **C4** | **F3** | **F4** |
| --- | --- | --- | --- | --- |
| Overall (both lists) |  |  |  |  |
| Slow spindles  List 1 | 0.33 | 0.39 | 0.32 | 0.26 |
| Slow spindles | -0.15 | 0.03 | -0.13 | -0.08 |
| List 2 |  |  |  |  |
| Slow spindles | 0.67** | 0.60 | 0.63* | 0.44 |
|  |  |  |  |  |

Pearson correlation values between contextual memory score for both lists (first row), first and second list and the number of slow spindles recorded at each of the four electrode sites. **P* < 0.05, ** *P* < 0.03.
